# Transformative Physiotherapy Approach in an 80-Year-Old Female: A Case Report of Managing Obstructive Sleep Apnea for Improved Quality of Life

**DOI:** 10.7759/cureus.57481

**Published:** 2024-04-02

**Authors:** Rishika Gabada, Vaishnavi Yadav, Dhanshri Nikhade

**Affiliations:** 1 Physiotherapy, Ravi Nair Physiotherapy College, Datta Meghe Institute of Higher Education & Research, Wardha, IND

**Keywords:** relaxation exercises, sleep disturbances, obesity, breathlessness, respiratory muscle training, obstructive sleep apnea

## Abstract

Obstructive sleep apnea (OSA) presents a significant challenge to patients’ overall health and well-being, characterized by upper airway collapse during sleep leading to fragmented and non-restorative sleep patterns. This case report describes an 80-year-old female patient presenting with breathlessness, obesity (BMI: 43 kg/m^2^), sleep disturbances, fatigue, attention deficits, reduced chest compliance, and a history of type 2 diabetes mellitus. Clinical findings revealed ongoing sleep disruptions, worsening breathlessness, progressive weakness, and decreased oxygen saturation levels. The therapeutic intervention involved a comprehensive physiotherapy program targeting respiratory muscle training, lung function improvement, peripheral muscle strengthening, and relaxation exercises. The discussion highlights studies supporting physiotherapeutic interventions such as thoracic extension exercises, neuromuscular stimulation, and oropharyngeal exercises for managing OSA symptoms. Overall, this case underscores the importance of tailored physiotherapy interventions in addressing the multifaceted challenges of OSA, aiming to improve patient outcomes and quality of life.

## Introduction

Arousal-induced sleep disturbances or decreased oxygen saturation are the results of partial or whole upper airway collapse, which is known as obstructive sleep apnea (OSA) [1]. This disturbance results in fragmented sleep that is not restorative. Other warning signs include loud snoring, breathing pauses during sleep, and an overwhelming inclination to feel tired during the day [2]. The pharynx’s constriction and closure during sleep is a complicated phenomenon that is impacted by several pathophysiological variables. Upper airway blockage during sleep is probably caused by a confluence of variables, including anatomical risk factors and reduced neuromuscular activity and ventilatory drive [3]. A wide range of pathophysiological factors influence the complex phenomenon of pharynx constriction and shutting during sleep. Reduced neuromuscular activity, ventilatory drive, and anatomical risk factors are likely contributing factors to upper airway obstruction during sleep [4]. Moreover, partial or complete airway collapse that occurs repeatedly can be caused by a decrease in upper airway muscular tone [5].

The primary three root causes of an upsurge in adult cases of OSA are obesity, male gender, and advancing age. When age is taken into consideration, the severity tends to decrease with BMI [6]. Significant ramifications arise from OSA, impacting not only an individual’s general quality of life but also their cardiovascular and mental health, as well as their safety when driving [7]. Anatomic factors are micrognathia, retrognathia, facial elongation, mandibular hypoplasia, adenoid and tonsillar hypertrophy, and inferior displacement of the hyoid, and nonanatomic risk factors are central fat distribution, obesity, advanced age, male gender, supine sleeping position, and pregnancy [8]. OSA is a prevalent ailment that can lead to serious negative outcomes [9]. The effects, which are characterized by five or more episodes per hour, affect around one billion people globally [10]. With 15 or more episodes per hour, moderate to severe OSA affects over 425 million adults between the ages of 30 and 69 [11]. Studies have shown that the structure of upper airway soft tissues and obesity are two risk variables that are influenced by genetic inheritance [12]. A combination of negative collapsing pressure during inhalation and considerable retropalatal narrowing during expiration, with the latter contributing progressively to the obstruction, is often responsible for sleep-related upper airway obstruction [13].

Upper airway obstruction associated with sleep is frequently caused by a combination of negative collapsing pressure during inhalation and significant retropalatal narrowing during expiration, with the latter contributing progressively to the obstruction [14]. Profound daytime sleepiness, loud snoring, and episodes of gasping, choking, or disrupted breathing during sleep are common symptoms in individuals with suspected OSA. Among these signs and symptoms, excessive daytime sleepiness is one of the most common [15]. For optimal outcomes, exercise therapy for OSA patients must be customized according to their specific requirements, taking into consideration their weight, nutrition, level of physical activity, and any underlying medical issues. To improve overall support, it is recommended to emphasize cardiovascular, respiratory, and oropharyngeal activities in addition to traditional treatments. Exercise training programs are especially advised since they have a good impact on the inflammatory profile of people with OSA [16,17].

## Case presentation

The 80-year-old female patient was brought in by her family with a primary complaint of breathlessness, which was grade 2 on the Modified Medical Research Council (MMRC) scale and had been getting worse day by day. The patient was diagnosed with OSA at an adjacent hospital. Along with the difficulty breathing, the patient also suffered from obesity with a BMI of 43 kg/m^2^, weight gain of approximately 110 kg, sleep disturbances that occurred after 1-1 hour, especially during the night, activities of daily living that included standing, dressing, undressing, etc., fatigue while walking, attention deficits that included forgetting things, names, etc., which were assessed with a mini-mental scale examination, reduced chest compliance, and a history of type 2 diabetes mellitus (DM) since 30 years on medication. Currently, the patient is on injections of Meropenem 500 mg, Pantop 40 mg, Perinorm 10 mg, Mucomix 600 mg, Hydrocort 100 mg, Tablet Dytor 10 mg, and Doxofylline 400 mg. Furthermore, she stated experiencing fever, burning micturition, and body swelling throughout the body.

Clinical findings

**Figure 1:**
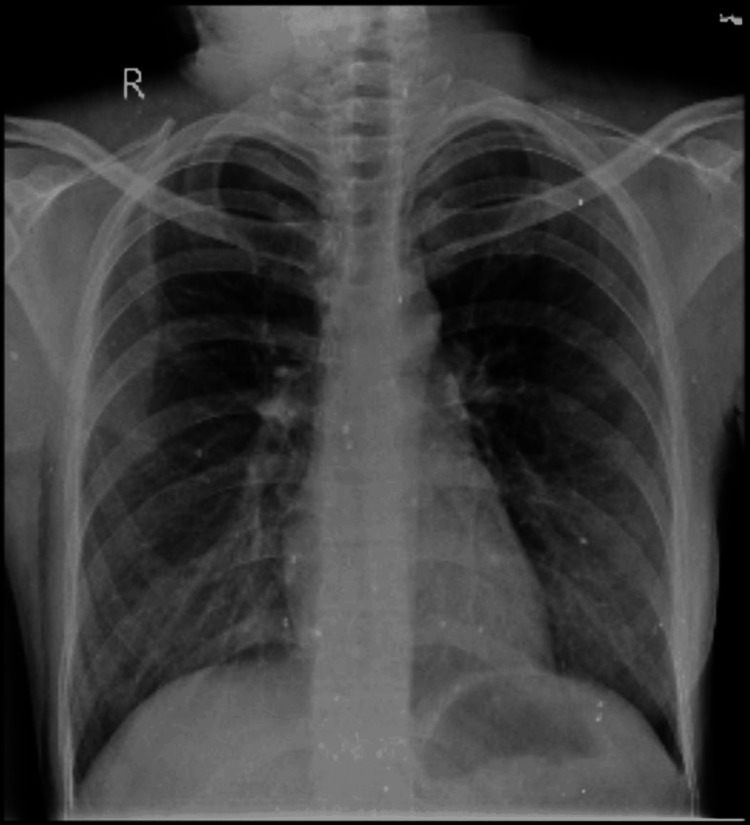
Chest X-ray Interpretation: heterogeneous opacity in the B/L lower and middle zones; prominent bronchovascular marking present in the middle and lower zones

The patient experienced ongoing sleep disruptions for one year, characterized by sleepless nights and disrupted sleep. She also struggled with breathlessness, which is grade 4 on the MMRC scale, which worsened daily, and a progressive loss of strength, which was measured through manual muscle testing and was grade 2/5, which made even daily tasks more difficult. The concerning issue of not sustaining saturation levels in the bloodstream, which led to an intricate interplay of symptoms, complicated these health issues, and the patient was on 6 L/O2. This complex network of health problems eventually had an unanticipated result: a weight gain of 110 kg, which also affected bed mobility. The body, overburdened by these many problems, experienced transformations that went beyond the physical and invaded the individual’s overall well-being. The complete incident’s timeframe is depicted in Table 1. The investigations that were conducted are shown in Table 2 and Table 3, respectively. The chest X-ray is shown in Figure 1.

**Table 1:** Timeline

Date	Event
10/11/2023	The patient visited the hospital.
11/11/2023	Investigations were done.
12/11/2023	The physiotherapy started.
20/11/2023	The patient was discharged.

**Table 2:** Blood investigations ALP, alkaline phosphatase; ALT, alanine aminotransferase; AST, aspartate aminotransferase; WBC, white blood cell

Investigation	Report finding	Normal value
Hb%	10	12-16 g/dL
Total WBC count	12,000	5,000-10,000/μL
ALP	112	45-115 U/L
ALT	17	7-55 U/L
AST	23	8-48 U/L
Total protein	6.3	6.0-8.3 g/L
Albumin	2.8	3.5-5.5 g/dL

**Table 3:** ABG test result Interpretation: partially compensated respiratory acidosis ABG, arterial blood gas

Component	Value
pH	7.303
pCO_2_	64.2
pO_2_	97.3
cHCO_3_	27.9

Therapeutic intervention

**Figure 2:**
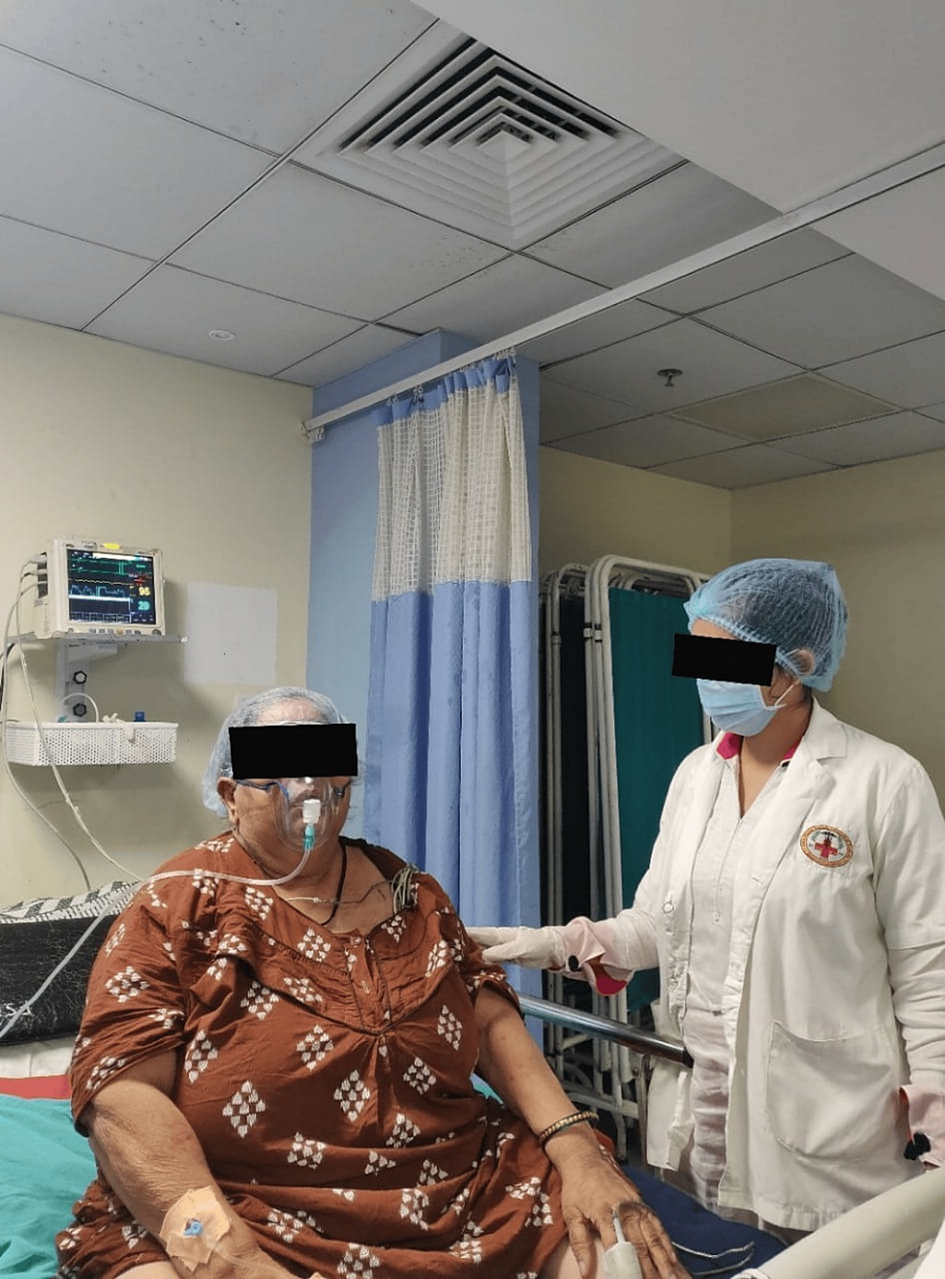
The patient performing pursed-lips breathing exercise

The physiotherapy protocol is given in Table 4. Physiotherapy sessions included respiratory muscle training, which included pursed-lips breathing, as shown in Figure 2.

**Table 4:** Physiotherapy intervention

Intervention	Description	Duration	Sets and repetitions
Patient and education relative	Explaining the importance of the information regarding their health and overall well-being		
Respiratory muscle training	Targeting inspiratory muscles, especially the diaphragm	Tailored to the patient’s state of fatigue, increasing in intensity as capacity improves	Pursed-lips breathing: two sets, 10 reps initially, progressing as tolerated
	Initial phase: introducing pursed-lips breathing exercises, focusing on nasal inhalation and slow exhalation	Gradual extension of exercises, incorporating postural variations and resistance for intensity	
Improving lung function	Enhancing overall respiratory efficiency	Integrated into the overall treatment plan, adjusted based on patient progress	Diaphragmatic and thoracic expansion exercises: two sets, 10 reps
	Ongoing exercises to maximize diaphragmatic and thoracic expansion		Adjusted based on the patient’s progress
Peripheral muscle strengthening	Reducing reliance on the shoulder girdle and neck muscles	Altered by the patient’s progress and tolerance and integrated into the overall treatment plan	Two sets, 12 reps
	Emphasizing positions that minimize reliance on shoulder and neck muscles		Incorporating resistance gradually
Relaxation exercises	Emphasizing relaxation techniques during breathing exercises	Regular practice, extending as needed for sustained impact	Emphasis on relaxation throughout breathing exercises: two sets, five minutes
	Continuing emphasis on relaxation throughout breathing exercises		Regular practice for ongoing benefit
			Incorporating relaxation techniques: three sets, 10 minutes
	Incorporating relaxation techniques from yoga for a holistic impact		

Follow-up and outcome

The pre-treatment outcome and post-treatment outcome are shown in Table 5.

**Table 5:** Outcome measures

Follow-up and outcome	Pre-treatment	Post-treatment
Hospital Anxiety and Depression Scale	10/21	7/21
WHOQOL	30/100	50/100
ICU bed mobility scale	1/10	5/10

## Discussion

The case report details the management of OSA in an 80-year-old female through tailored physiotherapy. The patient’s age, obesity, and type 2 DM posed unique challenges. The physiotherapy program, including respiratory and peripheral muscle training, led to significant improvements in the patient’s quality of life, as indicated by improved mobility and reduced anxiety and depression scores.

Eyuboglu et al. conducted a study in 2023 that concluded thoracic extension exercises (TY) were found to have significant beneficial effects on those with OSA syndrome (OSAS). The results of the study showed improvements in measures related to heart rate and inspiratory muscle strength during cardiopulmonary exercise testing. In addition, compared to a control group, OSAS patients who participated in TY showed improvements in neurocognitive performance, sleep efficiency, quality, and duration, as well as a decrease in daytime sleepiness [18].

According to a 2004 study conducted by Lequeux et al., OSAS could benefit from physiotherapeutic therapies that emphasize neuromuscular stimulation and muscular exercises. According to the study, this type of treatment could prove beneficial, particularly in mild cases, as it may prevent the progressive onset of muscle hypotonia linked to OSAS [19].

In 2020, Atilgan et al. conducted a study that indicated oropharyngeal exercises, when combined with posture and cervical area exercises, improved the patients’ general well-being, decreased their degree of fatigue, and increased the quality and functional capability of their sleep. As a result, it is thought that oropharyngeal exercises may be utilized as an additional OSAS treatment approach [20].

## Conclusions

This instance emphasizes the severe effects that OSA can have on individuals, particularly when combined with additional health conditions like obesity and type 2 diabetes mellitus. The treatment plan that was presented was extremely beneficial in reducing the variety of symptoms related to OSA and improving the patient’s overall quality of life. It included individualized physiotherapy interventions that focused on respiratory and peripheral muscle endurance. Heading into the future, continued exploration of innovative treatment modalities remains imperative to further optimize outcomes and enhance the well-being of individuals grappling with OSA.
